# Multimodal assessment of sustained threat in adolescents with nonsuicidal self-injury

**DOI:** 10.1017/S0954579421000754

**Published:** 2021-12

**Authors:** Zeynep Başgöze, Salahudeen A. Mirza, Thanharat Silamongkol, Dawson Hill, Conner Falke, Michelle Thai, Melinda Westlund Schreiner, Anna M. Parenteau, Donovan J. Roediger, Timothy J. Hendrickson, Bryon A. Mueller, Mark B. Fiecas, Bonnie Klimes-Dougan, Kathryn R. Cullen

**Affiliations:** 1 Psychiatry and Behavioral Sciences, University of Minnesota Medical School, Minneapolis, MN, USA;; 2 Department of Psychology, University of Minnesota, Minneapolis, MN, USA;; 3 Division of Biostatistics, School of Public Health, University of Minnesota, Minneapolis, MN, USA;; 4 Institute of Child Development, University of Minnesota, Minneapolis, MN, USA;; 5 University of Minnesota Informatics Institute, Minneapolis, MN, USA;; 6 Department of Psychiatry, Huntsman Mental Health Institute, University of Utah, Salt Lake City, UT, USA; 7 Department of Psychology, University of California, Davis, CA, USA

**Keywords:** adolescents, nonsuicidal self-injury, RDoC, sustained threat

## Abstract

Nonsuicidal self-injury (NSSI) is a common but poorly understood phenomenon in adolescents. This study examined the Sustained Threat domain in female adolescents with a continuum of NSSI severity (*N* = 142). Across NSSI lifetime frequency and NSSI severity groups (No + Mild NSSI, Moderate NSSI, Severe NSSI), we examined physiological, self-reported and observed stress during the Trier Social Stress Test; amygdala volume; amygdala responses to threat stimuli; and resting-state functional connectivity (RSFC) between amygdala and medial prefrontal cortex (mPFC). Severe NSSI showed a blunted pattern of cortisol response, despite elevated reported and observed stress during TSST. Severe NSSI showed lower amygdala–mPFC RSFC; follow-up analyses suggested that this was more pronounced in those with a history of suicide attempt for both moderate and severe NSSI. Moderate NSSI showed elevated right amygdala activation to threat; multiple regressions showed that, when considered together with low amygdala–mPFC RSFC, higher right but lower left amygdala activation predicted NSSI severity. Patterns of interrelationships among Sustained Threat measures varied substantially across NSSI severity groups, and further by suicide attempt history. Study limitations include the cross-sectional design, missing data, and sampling biases. Our findings highlight the value of multilevel approaches in understanding the complexity of neurobiological mechanisms in adolescent NSSI.

## Introduction

Nonsuicidal self-injury (NSSI), the deliberate harming of one’s own bodily tissue without the intent of suicide, is a prevalent and maladaptive behavior which often begins during adolescence (Brown & Plener, 2017). With a prevalence in adolescents of approximately 17% (Mars et al., 2019; Swannell, Martin, Page, Hasking, & St John, 2014), NSSI is associated with risk for negative outcomes including suicide attempts (Mars et al., 2019; Swannell et al., 2014). Typically, NSSI functions to relieve emotional distress (Klonsky, 2007). Few evidence-based interventions are available to address NSSI in adolescents (Glenn, Esposito, Porter, & Robinson, 2019; Schreiner, Klimes-Dougan, Parenteau, Hill, & Cullen, 2019; Turner, Austin, & Chapman, 2014). Advancement of new interventions will require new knowledge about the biological mechanisms underlying this complex behavior.

Since NSSI is a behavior that occurs across psychiatric diagnoses, a transdiagnostic approach in line with the National Institute of Mental Health’s Research Domain Criteria (RDoC) initiative holds promise for further understanding the underlying mechanisms of NSSI in adolescents. The RDoC framework integrates multiple levels of information (e.g., physiology, self-report, behavior, brain imaging) to examine dimensions of functioning relevant to psychopathology (Cuthbert & Insel, 2013). RDoC attempts to understand the whole spectrum of functioning, from adaptive to maladaptive, with the core assumption that measures from different levels of a given domain can be integrated to provide a fuller understanding of healthy and psychopathological functioning. The RDoC matrix is a tool to guide researchers in implementing this framework: rows represent domains of functioning (negative valence, positive valence, cognitive, social, arousal and regulatory, and sensorimotor) and subconstructs; columns represent levels of measurement (from genes to behavior).

While it is likely that dysfunction in multiple RDoC domains represents NSSI (Westlund Schreiner, Klimes-Dougan, Begnel, & Cullen, 2015), a highly relevant domain is Negative Valence, given that NSSI is most commonly used to relieve negative affect (Klonsky, 2007). Within this domain, the Sustained Threat construct is particularly relevant to NSSI. Adolescents who engage in NSSI typically report a combination of current and past (chronic) stressors (Guerry & Prinstein, 2010; Miller et al., 2018, 2019). The chronicity of NSSI and its association with early life stress, particularly childhood abuse, suggests the likelihood of neurobehavioral alterations in threat response which persist over the long term (Liu et al., 2014). Based on these considerations, the current RDoC study focuses on the Sustained Threat construct of the Negative Valence domain.

Sustained Threat is an aversive emotional state caused by prolonged exposure to stimuli that signal danger. Key neurobiological systems implicated in the response to threat include amygdala–frontal circuitry (Banks, Eddy, Angstadt, Nathan, & Phan, 2007; Hariri, Mattay, Tessitore, Fera, & Weinberger, 2003; Price & Drevets, 2012) and the hypothalamic–pituitary–adrenal (HPA) axis (Lupien, McEwen, Gunnar, & Heim, 2009; Zorn et al., 2017). Prior work has demonstrated developmental changes in Sustained Threat indices during adolescence. Fibers projecting from the amygdala to the prefrontal cortex continue to mature through adolescence and into adulthood (Gee et al., 2013; Perlman & Pelphrey, 2011; Swartz, Carrasco, Wiggins, Thomason, & Monk, 2014). Cross-sectional studies comparing adolescents to adults have shown that adolescents engage prefrontal regions to a lesser degree than adults when faced with threat imagery (Monk et al., 2003) and that the ratio of prefrontal/amygdala fear response increases across adolescence (Yurgelun-Todd & Killgore, 2006). Research examining physiological indices of the threat system has found that both the HPA axis and sympathetic nervous system responses to threat challenges are greater in mid-adolescence as opposed to late childhood/early adolescence, and that these indices are correlated with fearful temperament, anxiety, and depression symptoms in adolescents (Gunnar, Wewerka, Frenn, Long, & Griggs, 2009; Klimes-Dougan, Hastings, Granger, Usher, & Zahn–waxler, 2001; Stroud et al., 2009). Thus, a developmentally informed approach is critical to understanding the role of Sustained Threat in NSSI.

To date, research examining neurobiological correlates relevant to Sustained Threat in adolescents with NSSI has yielded mixed patterns. These studies have primarily been undertaken with single levels of analysis. To our knowledge, no studies have yet examined amygdala structure in relation to NSSI. Some (but not all) (Westlund Schreiner, Cullen, & Klimes-Dougan, 2017a, 2017b) functional magnetic resonance imaging (fMRI) studies have reported that individuals with NSSI demonstrate greater amygdala hyperactivity towards emotionally valenced stimuli than healthy controls (Niedtfeld et al., 2010; Plener, Bubalo, Fladung, Ludolph, & Lulé, 2012). We previously showed that adolescents with a history of NSSI exhibited atypical amygdala–frontal connectivity both during an emotion task and during rest (Westlund Schreiner et al., 2017b). Another recent study also demonstrated reduced amygdala–medial prefrontal cortex (mPFC) resting-state functional connectivity (RSFC) in adolescents with NSSI (Santamarina-Perez et al., 2019). Neuroendocrine studies have previously reported a blunted pattern of cortisol response to experimental stressors in adolescents with NSSI (Kaess et al., 2012; Plener et al., 2017), a finding that was also noted in a considerably larger sample of adolescents diagnosed with major depressive disorder comparing those engaging or not engaging in NSSI (Klimes-Dougan et al., 2019). Furthermore, these studies have yielded some suggestions of divergence in systems among adolescents with NSSI, manifesting as physiological blunting despite self-reported high levels of stress.

Recent multilevel work in the area of adolescent depression can highlight the potential promise of a multiple levels approach in adolescents with NSSI (Westlund Schreiner et al., 2017a). We have previously documented linkage between HPA and amygdala responses to threat in adolescents with and without depression (Klimes-Dougan et al., 2014). However, we have also found that when directly compared with healthy controls, adolescents with depression show different patterns of correspondence between HPA axis functioning, amygdala volume, and frontolimbic connectivity, suggestive of suboptimal coordination amongst systems in depression to efficiently rally and downregulate stress responses (Klimes-Dougan et al., 2014; Thai, Schreiner, Mueller, Cullen, & Klimes-Dougan, 2020). It could be that extended or sustained responses to threat in the context of chronic stress results in allostatic changes in one or more aspects of the stress response system (and/or in the coordination among different parts of the system), diminishing the ability to effectively regulate negative emotions. These insights helped to motivate the current work, suggesting that a careful examination of the interrelationship of measurements of the Sustained Threat construct across multiple units of analysis can reveal important new information about NSSI.

In the current study, we utilized an RDoC approach to examine the Sustained Threat domain using multiple levels of analysis in adolescents assigned female sex at birth who exhibit a continuum of NSSI severity (No, Mild, Moderate, and Severe). This current work focuses on cross-sectional (baseline) results on the Sustained Threat construct of the Negative Valence domain from an ongoing longitudinal study called the Brain Imaging Development of Girls’ Emotion and Self (BRIDGES) Study whose overarching goals are to examine domains of Negative Valence, Cognitive Control, and Self-Processing longitudinally in a sample enriched for NSSI (IRB #1605M881020). The decision in the BRIDGES study to focus on brain development in female adolescents was based on two factors: (a) NSSI is more common in females than males (Barrocas, Hankin, Young, & Abela, 2012) and (b) given the known sex differences in brain development and HPA axis (Giedd et al., 2006; Goddings et al., 2014; Klimes-Dougan et al., 2001), focusing on just one sex minimizes heterogeneity and improves statistical power to discover brain-behavior relationships within that sex.

The first aim of this current study is to test how different units of analysis of Sustained Threat (see Table 1) relate to NSSI severity. We hypothesized that multilevel measures of Sustained Threat would *individually* relate to NSSI severity. As an initial step we first explored the interrelationships between all the continuous variables used in this study (including NSSI lifetime frequency). We expected to observe meaningful relationships between NSSI severity and Sustained Threat measures. For example, based on our previous work (Westlund Schreiner et al., 2017b), we hypothesized that higher NSSI severity would correlate with lower amygdala–mPFC RSFC.

We then conducted analyses with a clinically meaningful categorical index of NSSI severity (defined using both frequency of NSSI and injury severity) to examine each Sustained Threat measure in relation to NSSI. Given the possibility that complex, nonlinear (e.g., allostatic) relationships may be present, we utilized this categorical approach as it takes into account both severity and injury, does not require an assumption of a linear relationship between NSSI severity and each measure, and allows for more interpretable visualization of how NSSI groups differ with respect to Sustained Threat neurobiology (especially when examining cortisol change over time). Thus, we expected to see different patterns of neurobiological responses in moderate versus severe NSSI groups.

Second, we hypothesized that the Sustained Threat measures from different levels would also relate to NSSI severity *together*. Here we used multi regression models with NSSI severity as a continuous outcome variable and the summary Sustained Threat measures as predictors, and we expected to observe that combinations of threat measures better predict NSSI severity together rather than on their own.

Third, we explored how the interrelationships among Sustained Threat measures vary by NSSI severity by comparing the correlation patterns between multilevel threat measurements across different NSSI severity groups. We hypothesized that, due to allostatic processes that may occur over the course of NSSI progression, the different NSSI severity groups would exhibit different interrelationship patterns. In addition, we applied follow-up analyses to test whether any significant relationships with NSSI could also be explained by other clinical and demographic factors such as comorbid depression, past trauma, anxiety, age, and medication status. Finally, given that NSSI is a known risk factor for suicide attempts, and that aberrant patterns of Sustained Threat may also underlie risk for suicidal behavior, we also explored relationships with suicidality.

## Method

### Ethics and oversight

The study was approved by the Institutional Review Board at the University of Minnesota. Although this longitudinal study is not a clinical trial, it was registered in ClinicalTrials.Gov, NCT02947308. Data and safety monitoring were provided by the University of Minnesota Center for Translational Science Institute.

### Inclusion criteria

We recruited adolescents who were assigned as female sex at birth, between 12–16 years old with and without a history of NSSI to participate in a longitudinal investigation. The current work is largely based on the data collected at the first assessment. In four cases, data from the Time 2 visits were used because the MRI visit was not completed at Time 1, making the age range for the current sample 12–17 years. The study was oversampled for NSSI behaviors, and participants represent a range of severity in NSSI engagement. In particular, we aimed to recruit four equally sized groups of NSSI severity: no NSSI, mild NSSI, moderate NSSI, and severe NSSI (methods for assigning these groups are described below). Participants were excluded if they had a history of current substance use disorder, lifetime bipolar disorder or psychotic disorder, any intellectual or developmental disability, any major medical illness that would impact brain structure or function, or any contraindications to undergoing magnetic resonance imaging (including having braces) at intake.

### Recruitment, screening and consent

Participants were recruited through flyers posted in the community, digital marketing, and local clinics and hospitals. Following a telephone screen to review inclusion/exclusion criteria, participants and their legal guardians were invited to participate in an initial in-person session to complete the informed assent (adolescent) and consent (legal guardian) process and a diagnostic clinical interview to determine eligibility. During the COVID-19 pandemic, this took place via teleconference.

### Study structure

The longitudinal study enrolled participants from December 2016 through June 2020. The current paper focuses on the data from the Time 1 visit (although as noted above Time 2 data were used for four participants). At the time of writing, longitudinal data collection is ongoing. Study activities consist of three visits which occur annually: (a) consent and clinical assessment; (b) the Trier Social Stress Test (TSST; Kirschbaum, Pirke, & Hellhammer, 1993); and (c) neuroimaging. Because of the large number of self-report measures, some were completed on the first clinical visit and others were sent to the participants electronically to complete on their own time.

### COVID-19 impact

Of note, this study experienced some disruption due to the COVID-19 pandemic. Prior to the onset of the pandemic, all study visits were conducted in person, and were scheduled to take place around the same time (within a period of 2–3 weeks). In March 2020, all in-person research was shut down at the University of Minnesota for several months. At that time the study shifted the consent procedures and clinical interviews to a virtual platform. Neuroimaging and TSST visits were temporarily halted. In July 2020, the study resumed neuroimaging visits at a limited rate, utilizing extensive procedures to ensure safety and protection of participants and staff from the spread of infection of COVID-19. We decided not to resume in-person TSST visits due to safety concerns during the pandemic. We converted the TSST procedure to a virtual platform in October 2020; however, the current paper focuses only on the available data collected from the in-person Trier visits. Because of these disruptions, study visits for some participants were spaced apart by several months. Once neuroimaging resumed, given constraints that prevented rapid catch-up, our strategy in scheduling catch-up scans was to prioritize the minimization of spacing, with an absolute threshold of 6 months, beyond which we postponed scanning until the following year in the longitudinal assessment. To address the issue of asynchronicity, we repeated select clinical measures.

### Clinical assessment

Participants and their guardian completed the clinician-administered semi-structured diagnostic interview, Kiddie Schedule of Affective Disorders and Schizophrenia Present and Lifetime Version (KSADS-PL) (Kaufman et al., 1997) to evaluate psychiatric conditions and to further assess eligibility. The paper version (KSADS-PL) was administered to 23% of the sample (*N* = 37) and the computerized version (KSADS-COMP) (Townsend et al., 2020) was administered to the remainder of the sample. The clinician-administered Self-Injurious Thoughts and Behaviors Interview (SITBI) (Nock, Holmberg, Photos, & Michel, 2007) was also administered to the adolescents during the intake visit to measure NSSI frequency in the past month, past year, and lifetime; average age of onset; severity of injuries (number of injuries per episode, severity of tissue damage – worst point and average); and other information regarding suicidal thoughts and behaviors. In cases where there were more than 2 months between the intake visit and MRI scan due to COVID-19, we repeated the SITBI either on or around the day of the MRI scan. Parents reported on key demographic variables including sex assigned at birth, ethnicity, race, and income. Participants completed self-report questionnaires at the end of the initial clinical interview visit. These included the Beck Scale for Suicidal Ideation (BSSI) (Beck, Kovacs, & Weissman, 1979), a 19-item instrument measuring current suicidal ideation, with ratings for active suicidal desire, specific suicide plans, and passive suicidal desire; the Beck Depression Inventory – Revised (BDI-II) (Beck, Steer, & Brown, 1996), a 21-item questionnaire assessing depressive symptom severity in the past two weeks; and the Child Trauma Questionnaire (CTQ) (Bernstein, 1998), a 28-item instrument assessing lifetime emotional abuse, physical abuse, sexual abuse, emotional neglect, and physical neglect. The current analyses used the total CTQ score representing a summary of all subscales of adverse experiences. Participants completed other self-report questionnaires through online administration on a separate day, which included the Personality Assessment Inventory Adolescent Form (PAI-A) (Morey, 2007). The current analyses focused on the anxiety subscale (PAI-A anxiety).

### NSSI outcomes

The SITBI was used to generate the NSSI severity outcome variables used in subsequent analysis. For our dimensional NSSI outcome we used the total number of lifetime NSSI episodes. For the categorical grouping we initially created four groups based on both number of episodes and on severity of injury: No NSSI, Mild NSSI (fewer than four past episodes involving significant tissue damage, or unlimited NSSI episodes with no tissue damage); Moderate NSSI (four or more past NSSI episodes, with frequency less than once per month, and with significant tissue damage); and Severe NSSI (four or more past NSSI episodes, with frequency greater than once per month, and with significant tissue damage). This grouping criterion was mainly based on the approach initially proposed in the KSADS in which fewer than four episodes of NSSI lifetime episodes was determined to be a subclinical level of engaging in NSSI (Kaufman et al., 1997). We have further validated this approach in a recent study showing a stepwise functioning of more blunted HPA response to threat in adolescents who engage in NSSI (most blunted for those with more than four episodes, moderate blunting for those with subclinical NSSI, and a normative stress response shown for healthy adolescents) (Klimes-Dougan et al., 2019). Other studies have used similar indexes of NSSI cut offs (Esposito-Smythers et al., 2010) and shown clinical utility (Muehlenkamp, Brausch, & Washburn, 2017). In this way, we could account for a more complete definition of NSSI that included both frequency and severity of injury.

As described below, since the sample size of the Mild NSSI group was very small (*N* = 14), we collapsed the No NSSI and Mild NSSI groups together for greater statistical power. In addition, the SITBI was used to measure the history of suicide attempt(s).

### Trier social stress test (TSST): Cortisol reactivity, reported and observed stress

The modified TSST is a laboratory procedure in which participants are asked to give a class introduction speech and perform verbal arithmetic calculations, each for five minutes, while being observed by two examiners who wear white lab coats and are trained to remain neutral and to avoid giving reassurance or feedback (Kirschbaum et al., 1993). Five cortisol measurements are collected during the procedure: CORT1 pre-task, CORT2 immediately after the speech and math section (+15 minutes), CORT3 at +30 minutes, CORT4 at +45 minutes, and CORT5 at +60 minutes. For each saliva sample, participants pushed their saliva through a straw and into a 1.5 ml vial (passive drool method). Samples were labeled and stored in a −25 °C freezer until they were shipped to Universitat Trier in Trier, Germany, for analysis. Researchers used assay methods consistent with Dressendörfer, Kirschbaum, Rohde, Stahl, & Strasburger, 1992. Cortisol values at three standard deviations above the mean were winsorized to be within three standard deviations of the mean for all analyses.

### Summary indices of cortisol values

In addition to the five individual cortisol values (CORT1, CORT2, CORT3, CORT4, CORT5) measured over the course of the TSST, we also computed the area under curve with respect to increase (AUCi), and used it as the primary summary index for physiological response following prior work (Klimes-Dougan et al., 2019). This index assesses the relative increase in cortisol evoked by the stressor irrespective of basal level prior to the task (Pruessner, Kirschbaum, Meinlschmid, & Hellhammer, 2003) and was assessed as a measure of cortisol reactivity.

### Observed and self-reported stress

In addition to cortisol measures, the modified TSST procedure included measures of observed expression of stress (examiner ratings) and participant self-reported experience of stress. To rate observed stress, during the TSST, the two examiners independently responded to the questions, “How stressed did the participant appear during the storytelling task?” and “How stressed did the participant appear during the arithmetic task?” on a scale from 1 (*not stressed at all*) to 5 (*considered discontinuing the procedure because they looked so stressed*). Examiner ratings were moderately correlated, *r*(112) = .697, *p* < .001. The two examiner ratings were averaged together for each of the math and speech tasks. To rate the experience of stress, after completion of the TSST, participants responded to the questions, “How stressful was giving the speech (class introduction)?” and “How stressful was the subtraction task?” on a scale from 1 (*calm*) to 5 (*high stress*). For an understanding of the temporal nature of self-reported stress, participants were also asked “How stressful was the period of time when you were preparing your speech (class introduction) and thinking about what to say?” and “How do you feel now?” A mean stress score across speech and math tasks were used as the summary score, respectively for observed and reported stress. Self-reported stress during the preparatory period and immediately after the arithmetic section were not included in the summary score, although they were included in analyses of group × time differences in stress trajectory.

### MRI data acquisition

Brain scanning sessions were conducted at the Center for Magnetic Resonance Research at the University of Minnesota using a Siemens 3 Tesla Prisma scanner (Erlangen, Germany) and a 32-channel receive-only head coil, using the Human Connectome Project (HCP) (UpAndRunning, 2021) multiband sequences to collect high spatial and temporal resolution fMRI data. Structural scans were acquired using an eight-minute T1-weighted, multiecho MP-RAGE (Magnetization-Prepared RApid Gradient-Echo) sequence with following parameters: Repetition time (TR) = 2,500 ms, echo times (TE) = 1.81/3.60/5.39/7.18 ms, inversion time (TI) = 1,000 ms; 0.8 mm isotropic voxels, field of view (FOV) = 256, flip angle = 8 degrees and a six-minute T2-weighted SPACE (Sampling Perfection with Application optimized Contrasts using different flip angle Evolution) sequence: TR = 3,200 ms, TE = 564 ms, 0.8 mm isotropic voxel, FOV = 256, variable flip angle. Two HCP spin Echo Planar Imaging (EPI) field map scans (anterior–posterior [AP] and posterior–anterior [PA] phase encode, 1 minute total) were acquired with voxel parameters matching those of the fMRI task acquisition and were used to correct the fMRI data for the geometric distortion caused by magnetic field inhomogeneity.

Next, a series of functional fMRI scans were obtained consisting of whole brain T2*-weighted functional volumes with 2 mm isotropic voxel resolution, with the following task fMRI parameters: TR = 800 ms, TE = 37 ms, flip angle = 52°, FOV = 212 mm, 2 mm isotropic voxel, Multiband factor=8. All functional data are acquired using the HCP multiband echo planar imaging sequence. The first of these consisted of a 12-minute (912 volume) resting-state scan during which participants were instructed to stay awake, keep their eyes open focused on a fixation cross, and to “not think about anything in particular.” Participants then completed the threat (an emotion-face matching) task, described below. Subsequent neuroimaging acquisition that was collected in this study and not reported on here included an emotion GoNoGo task, a self-evaluation task, and two diffusion imaging sequences. Total scanning time for each MRI session was approximately 1.5 hours.

### Emotion face-matching task

Participants completed a task in the scanner (Hariri, Tessitore, Mattay, Fera, & Weinberger, 2002). The task was implemented using E-prime software (Schneider, Eschman, & Zuccolotto, 2002). Task stimuli were projected onto a screen inside the bore of the scanner using a mirror. For the matching of emotion faces condition, stimuli consisted of black-and-white photographs of human faces depicting anger and fear (Ekman & Friesen, 1976). For the control condition, stimuli consisted of horizontal and vertical ellipses of neutral colors. Participants were instructed to look at the picture in the top row and, using a response box, they selected one of the two pictures in the bottom row that matched the top row. In the control block, they were asked to match the shapes and in the affective block they matched the emotions of the faces. The task consisted of 13 counterbalanced blocks, 24s each: five face-matching blocks, five shape-matching blocks, and three blocks where participants viewed a fixation cross. This well-established task has been widely used to probe threat-related amygdala reactivity (e.g., Gerin et al., 2019; Nikolova et al., 2014; Prather, Bogdan, & Hariri, 2013; Swartz, Knodt, Radtke, & Hariri, 2015), because the facial expressions of negative and high arousal affect (such as the fear and anger faces in the task) prompt strong visceral responses in the body (Öhman & Soares, 1998), as well as robust engagement of the amygdala in the brain, all being the signatures of the threat mechanism (Hariri et al., 2002). The task has demonstrated reliability (Manuck, Brown, Forbes, & Hariri, 2007). The fMRI task acquisition lasted 6h 45min and contained 494 brain volumes.

### Neuroimaging data preprocessing

As described in detail in Supplementary Materials, HCP pipelines were used to process the neuroimaging data (Glasser et al., 2013).

### Addressing subject motion

Subject motion during scanning was calculated using framewise displacement, and volumes with displacement greater than 0.5 mm were flagged for excess motion (Power, Barnes, Snyder, Schlaggar, & Petersen, 2012). Datasets (per scan) with greater than 30% flagged volumes were excluded from analysis.

### Defining neuroimaging outcomes

For all imaging data, in accordance with our aims to study multiple levels of the Threat system, analyses focused on amygdala (Adolphs, 2008; Ohman, 2005) with respect to its volume, activation in response to threat task, and resting-state functional connectivity with the mPFC (Little & Carter, 2013; Phelps, Delgado, Nearing, & LeDoux, 2004; Quirk, Likhtik, Pelletier, & Paré, 2003).

### Structural

To measure amygdala volume, we extracted the gray matter volumes of right and left amygdala using Freesurfer’s automated subcortical segmentation tool (Fischl et al., 2002).

### Task fMRI

FSL FEAT (FMRI Expert Analysis Tool; Woolrich, Ripley, Brady & Smith, 2001) was used to conduct a regression analysis measuring neural activation at each parcel of the brain during the emotion matching task. We included two explanatory variables (emotion face and shape conditions). Our contrast of interest was emotion > shape. The produced emotion > shape *z*-score statistical maps in matrix form were projected back to CIFTI space for further analyses. Average *z*-scores for gray ordinates within left and right amygdala for the emotion > shape contrast were extracted for further analyses.

### Resting state

The CIFTI-space gray-ordinate-wise time series were used to create average time series for each of the Glasser and Harvard-Oxford parcellations. Then the average time series from bilateral amygdala and mPFC were extracted, cross-correlated, and Fisher’s *z*-transformed to yield *z*-scores representing the connection between amygdala and mPFC. The mPFC region of interest contains thick and lightly myelinated regions in the medial part of each hemisphere including areas of the anterior cingulate cortex as well as regions lying outside the cingulate cortex superiorly, anteriorly, and inferiorly (Glasser et al., 2016).

### Statistical analysis

We first conducted preliminary analyses on the demographic and clinical data to characterize the different NSSI groups. We examined the distribution of all measures to determine appropriateness for different analysis approaches.

Our overarching goal was to examine neurobiological correlates of NSSI using an RDoC approach with multiple levels of analysis in a transdiagnostic sample. Therefore, we specifically sought to test (1a) how summary measures of Sustained Threat are linked with biological indices and NSSI severity, (1b) if these Sustained Threat and biological indices would differ across NSSI severity groups, (2) if combinations of these measures would explain variance in NSSI severity better than a single threat measurement or a combination of psychological assessments, (3) how these measures related to each other across NSSI severity groups, testing the RDoC assumption that multiple levels of information from a given construct can be meaningfully integrated.

Consistent with these purposes, we considered NSSI severity as a continuous outcome for the Aims 1a and 2, and as a categorical outcome for the Aims 1b and 3. The continuous outcome variable was defined as the lifetime number of NSSI episodes. To address skewness, this variable was log transformed. For the categorical variable, our initial goal was to create four groups of NSSI severity as determined by the SITBI. However, the sample size of the mild NSSI group was significantly lower than the other groups (*N* = 14). Therefore, in order to balance the sample size across groups, we divided our sample into three groups: No + Mild NSSI (*N* = 51 + 14), Moderate NSSI (*N* = 57), and Severe NSSI (*N* = 42). In this case, the No + Mild NSSI group included adolescents both with and without psychiatric disorders, none of whom have demonstrated a pattern of repeated (four or more) episodes of NSSI.

Given that our sample (enriched for NSSI) represents a group of adolescents at elevated risk for future suicide attempts, we also explored how our multilevel threat measurements would relate to past suicide attempt(s) (SA) and suicide ideation in our adolescent participants, within the framework of NSSI severity. To explore how suicidal ideation can be predicted by our Sustained Threat measures, we used BSSI scores as a dimensional outcome. Furthermore, to evaluate SA in the context of NSSI, we created five composite NSSI/SA groupings: No + Mild NSSI without SA (*N* = 61), Moderate NSSI without SA (*N* = 31), Moderate NSSI with SA (*N* = 22), Severe NSSI without SA (*N* = 16), and Severe NSSI with SA (*N* = 25). Due to the low number of No + Mild NSSI group members with a history of SA (*N* = 3), we excluded this group in these analyses. We repeated the same analyses we applied to our main NSSI groups on these five composite NSSI/SA groups.

The following key variables were considered for our multilevel threat system analysis (see Table 1): (a) TSST participant self-reported stress; (b) TSST experimenter-observed stress; (c) the physiological response to stress as measured by TSST cortisol responses; (d) right and left amygdala volumes as structural indicators, (e) right and left amygdala activation during the emotion > shape condition of the emotion matching task; and (f) right and left amygdala–mPFC RSFC.

#### Aim 1a.

To explore how NSSI severity, suicidal ideation, Sustained Threat measures, and age relate to each other, we first conducted a correlation analysis including all continuous variables included in our study.

#### Aim 1b.

To pursue our question of how each of the Sustained Threat measures specifically relates to NSSI severity, we first conducted a series of separate one-way analyses of variance (ANOVAs) to compare NSSI severity groups with respect to each of our Sustained Threat measures. In addition to our summary cortisol variable (AUCi), which we used as a predictor in the regressions, examination of change in cortisol over time provides a more nuanced assessment of stress response dynamics. Thus, we applied linear mixed effects modeling to separately evaluate differences in change over time in self-reported, observed, and cortisol stress reactivity between NSSI groupings. Follow-up analyses explored the same temporal patterning applied to the five NSSI/SA groupings.

#### Aim 2.

To further understand how each of the Sustained Threat variables helps to explain the variance of NSSI severity and suicidality, we conducted a series of multiple linear regression models, using *continuous* NSSI and BSSI as our outcome variables, and our multilevel Sustained Threat variables as predictors. These models were then compared with respect to Akaike information criterion (AIC). First, we conducted a stepwise model testing, where the scope of testing included every possible combination of variables, between an intercept-only model and a model including all Sustained Threat variables. We then selected the model with the lowest AIC, i.e., the model composed of the combination of variables that together, best explained the variance of NSSI severity. Of note, the number of subjects included in the model selection process was smaller because we required data to be present on all variables. We then tested how much the variables in this “best explanatory” model could predict NSSI severity by applying linear regression models in a larger sample (since the number of variables in the “best” model is low, we could run our linear regressions in a larger pool of participants who had complete data for all these variables). We then compared this model to a model including only the covariates to see whether these specific variables had a better predictive power than the covariates, which are proven to be highly correlated with NSSI severity.

Follow-up analyses tested whether any significant effects describing relationships between Sustained Threat measures and NSSI or suicidality could be explained by other factors such as age, early childhood trauma experiences, depressive symptoms, anxiety, and the medication our participants were taking at the time of assessment. Therefore, we controlled our most explanatory models for age, BDI, CTQ, PAI-A anxiety, medication status, and reported how these adjustments changed these models’ significance in predicting our outcome variables.

#### Aim 3.

To examine how the coordination between multiple levels of the threat system may vary by NSSI severity, we created correlation matrices for each NSSI group representing the interrelationships of all of our Sustained Threat variables. The differences between correlation matrices were calculated using the Jennrich test of the equality of two matrices (Jennrich, 1970), and the *p* values were adjusted using false discovery rate (FDR) (Benjamini & Hochberg, 2000). We then adjusted our variables for age, CTQ, BDI, PAI-A anxiety, and medication status and reported how these adjustments changed the interrelations between the variables.

As a follow-up, we also created correlation matrices for all five of the NSSI and suicidality combined groups to see how suicidality affects the interrelationships of our variables across the NSSI groups, and tested their differences.

### Treatment of missing data

Different rates of missingness across different variables led to analytic challenges for application of imputation methods. Therefore, instead of imputation, we used all possible data available for each analysis. This led to smaller sample sizes for analyses that involved larger numbers of variables (degrees of freedom noted for each analysis in the results).

All statistical analyses were conducted in R (R Core Team, 2015). Figures were produced using the packages ggplot2 (Wickham, 2009) and ggcorrplot (Kassambara, 2016).

## Results

### Participants

**Figure 1.**
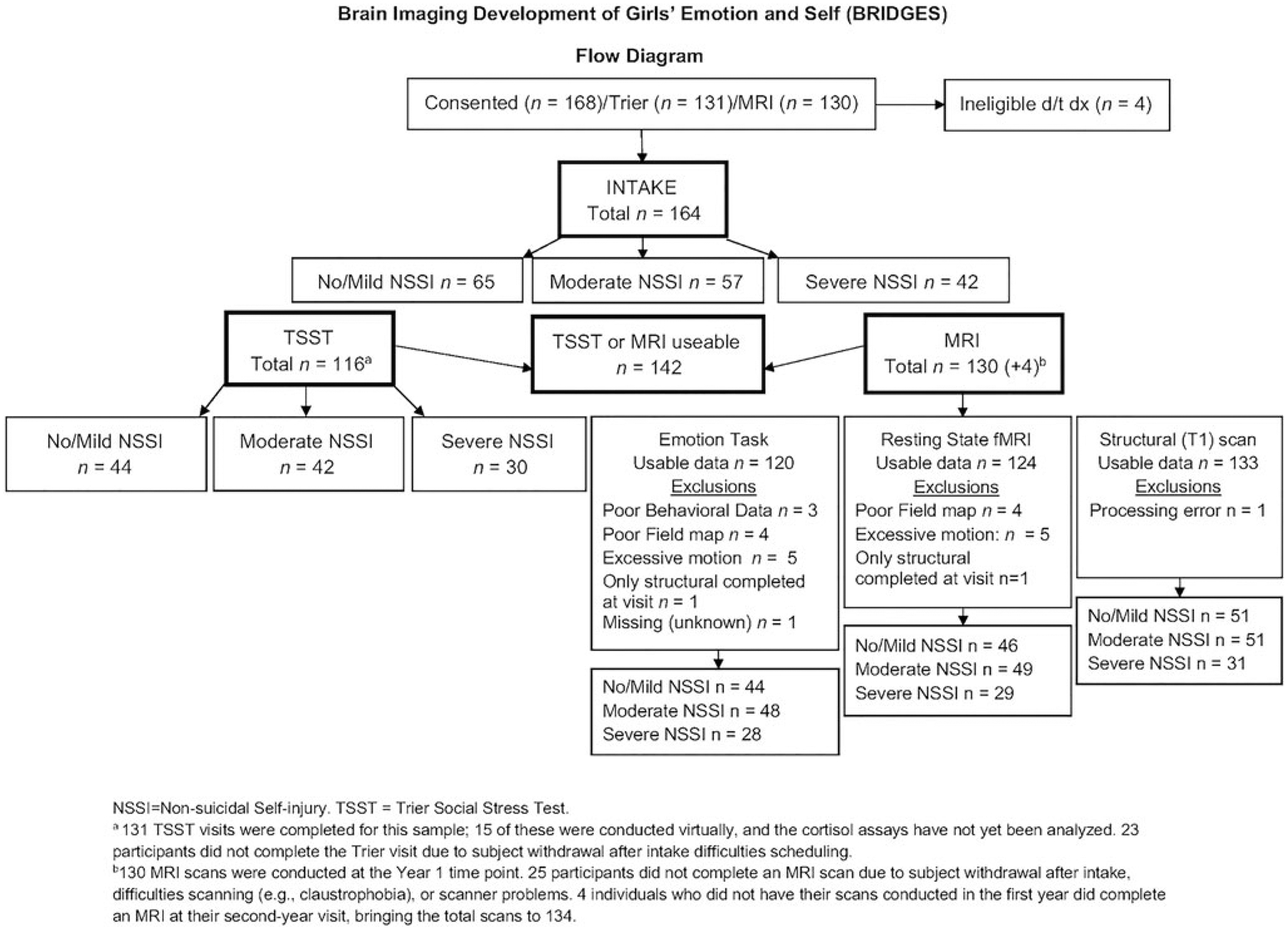
Flow diagram summarizing the relevant activities completed by participants in the Brain Imaging Development of Girls’ Emotion and Self (BRIDGES) study.

Figure 1 displays a flow diagram summarizing the activities completed by all participants in this study, capturing missing data and dropout. One hundred and sixty-eight adolescents completed consent and at least some of the assessment procedures (23 of the assessments were done via video conference due to the COVID-19 pandemic). Four participants were excluded based on diagnostic findings from the KSADS; 164 met inclusion criteria and were enrolled in the longitudinal study. Six participants withdrew from the study after the initial intake without completing any further procedures, and an additional 17 participants passively dropped out of the study at Time 1. Of the eligible participants, 137 completed at least one other baseline visit (TSST and/or MRI). One hundred and thirty participants completed the Time 1 neuroimaging session. Other participants did not complete the visit due to a number of reasons such as discomfort completing in-person visits during the COVID-19 pandemic, hospitalizations, and other difficulties in the scanner (e.g., claustrophobia). Four participants who did not complete their Time 1 neuroimaging session completed their first neuroimaging session during their Time 2 appointment. For those four participants, we included their Time 2 data with the analyses, making the total number of 134 completed MRI scans. For the emotion task, four participants did not have usable MRI data due to acquisition error, five participants were excluded due to excessive subject motion during the task fMRI scan, and three participants were excluded because their task accuracy was less than 50% (indicating poor engagement or possibly falling asleep). For the resting-state data, four participants were excluded due to acquisition error and five participants were excluded due to excessive subject motion during the resting fMRI scan. Thus, we had usable MRI data for 134 participants for structural data, 120 participants for the emotion task data, and 124 participants for the resting-state fMRI. One hundred and sixteen participants completed the in-person TSST procedure; data from all these subjects were included. One hundred and forty-two participants had *either* MRI *or* TSST data that are usable. Note that throughout the following sections, because of variability in missingness, the sample sizes change depending on which variable combinations are used in the analyses.

Table 2 summarizes the clinical and demographic data for all participants with either TSST AUCi cortisol or at least one of the imaging measures (amygdala volume, amygdala–mPFC connectivity, amygdala activation), separated by NSSI lifetime severity group (No + Mild, Moderate, and Severe), and documents the number of participants with usable data for each measure. Also see Table S2 in the Supplementary Materials for the clinical and demographic information for the five NSSI/SA groups described above.

### Preliminary analyses comparing groups on clinical and demographic variables

One-way ANOVAs demonstrated several expected group differences with respect to psychopathology. Results are summarized in the second section of Table 4. In summary, the Severe NSSI group had the highest BDI and PAI-A anxiety scores compared to other groups, whereas the Moderate NSSI group had the highest CTQ score. Furthermore, the Severe NSSI group showed a trend-level significance towards being older than the other groups. The groups did not differ from each other with respect to income. We further explored psychopathology across the NSSI severity/SA groupings. Results are summarized in Table S2, in the Supplementary Materials.

### Aim 1a: Examining the links between continuous measures of NSSI and BSSI, Sustained Threat measures, clinical measures, and age

We first examined the correlations between all of the variables in this study for the entire sample (see Table 3). As expected, we observed significant moderate positive correlations across clinical variables (depression and anxiety symptoms, childhood trauma experience and depressive symptoms, medication status and depressive symptoms, medication status and anxiety, NSSI severity and suicidal ideation, NSSI severity and depressive symptoms, NSSI severity and medication status). Pertaining more specifically to the aims that address the relationship between NSSI and the sustained threat, as predicted, lifetime NSSI frequency was found to be weakly negatively correlated with left and right amygdala–mPFC RSFC. However, contrary to predictions, for the whole group there was no evidence of significant associations between lifetime NSSI frequency and amygdala volume, activation, AUCi cortisol during the TSST, experience of stress during the TSST or observed stress during the TSST. Childhood trauma experience also showed a significant moderate negative correlation with right amygdala–mPFC RSFC, and a weak negative correlation with left amygdala–mPFC RSFC. Furthermore, we observed a significant but weak negative correlation between left amygdala activation during the emotion task and observed stress during the TSST.

### Aim 1b: Comparing NSSI severity groups on each Sustained Threat variable individually

A series of ANOVAs were conducted to compare the NSSI severity groups on each of the Sustained Threat variables listed in Table 1. We followed up these analyses with repeated testing that included relevant covariates which might contribute to the relationship between NSSI and Sustained Threat variables: age, BDI, CTQ, PAI-A anxiety, and medication status. Results are reported in Table 4. Of note, the follow-up analyses included smaller sample sizes because of missing values of the covariates (see Table 2).

NSSI severity groups significantly differed from each other on summary self-reported stress scores during the TSST. The Severe NSSI group reported higher stress compared to other groups; Moderate NSSI group reported higher stress than No + Mild NSSI, and lower stress than Severe NSSI. Although there was not a significant overall effect of group for AUCi and observed stress, pairwise comparisons suggested that AUCi was lower for Severe NSSI than the other groups, and that observed stress ratings were higher for Severe NSSI than both of the other groups. These group differences were no longer significant after adjusting for age, BDI, CTQ, PAI-A anxiety, and medication status.

**Figure 2.**
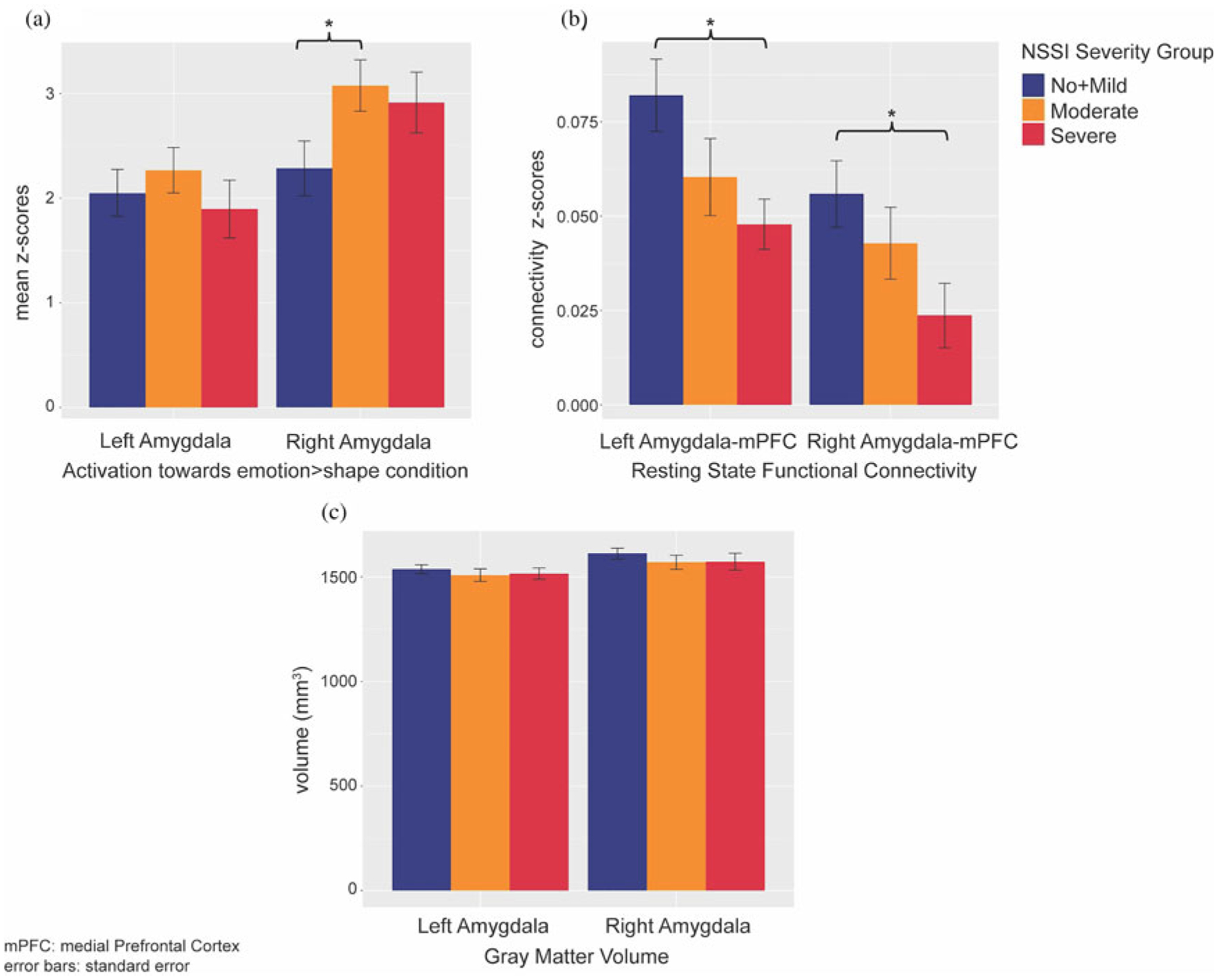
Nonsuicidal self-injury (NSSI) severity group differences of sustained threat brain measurements: (a) group differences of amygdala activations towards emotion > shape condition in emotion matching task; (b) group differences of amygdala–medial prefrontal cortex (mPFC) resting state functional connectivity; (c) group differences of amygdala gray matter volumes.

Amygdala volumes were highly similar across groups (Figure 2c); no significant or trend effects were observed, either with or without controlling for the covariates noted above. For amygdala activation, although the main group difference was not significant, pairwise comparisons suggested that right amygdala activation was higher in Moderate NSSI than No + Mild NSSI (Figure 2a). This group difference was no longer significant when the additional covariates noted above were included. A trend-level group difference was found for left amygdala–mPFC RSFC. Pairwise comparisons revealed that compared to No + Mild NSSI, Severe NSSI showed lower left and right amygdala–mPFC RSFC (Figure 2b). After controlling for age, BDI, CTQ, PAI-A anxiety, and medication status, previously trend-level-significant left amygdala–mPFC RSFC group difference lost significance, yet right amygdala–mPFC RSFC appeared to be significantly predicted by this combination of NSSI grouping and our covariates.

Results from follow-up analyses on the differences of our Sustained Threat variables across the five NSSI/SA groups are reported in the Supplementary Materials.

### TSST measurements over time: NSSI severity groups

**Figure 3.**
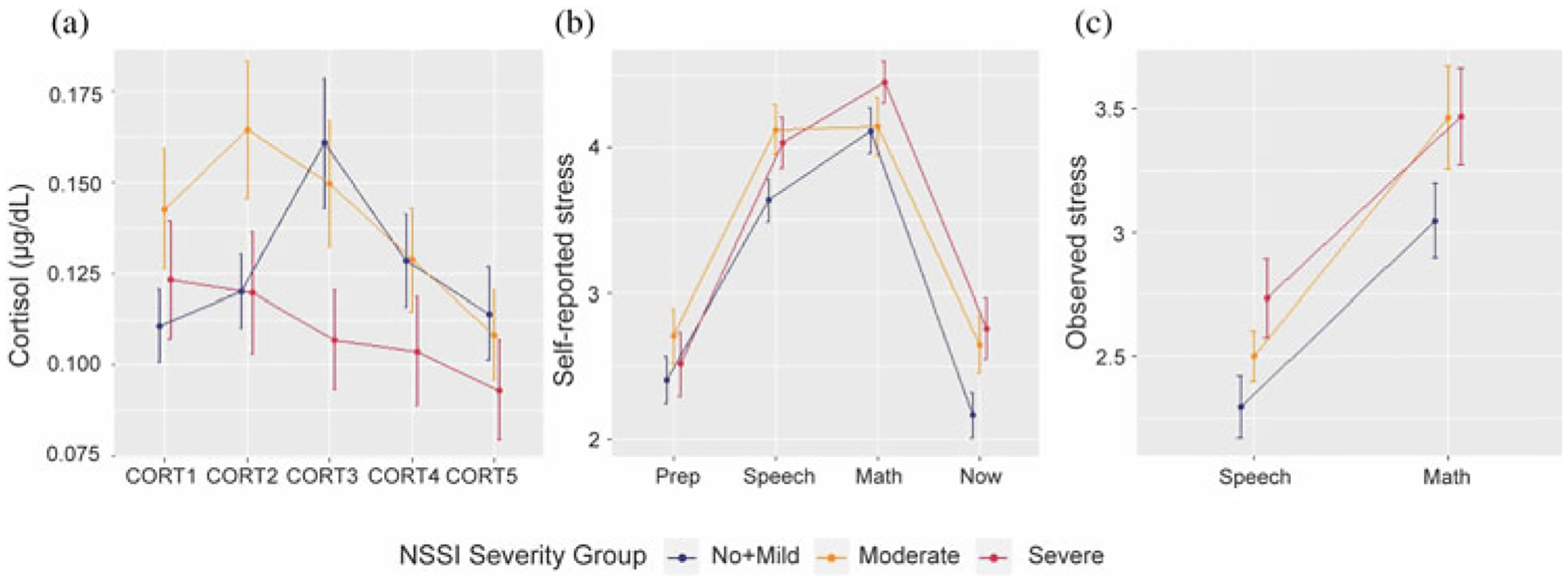
Stress measurement changes over time for three nonsuicidal self-injury (NSSI) severity groups: (a) cortisol temporal trajectory for different NSSI groups; (b) reported stress temporal trajectory for different NSSI groups; (c) observed stress temporal trajectory for different NSSI groups.

Linear mixed effects models on the cortisol levels over five points of time revealed a significant difference between NSSI severity groups, when we simultaneously tested the group and group by time interactions (*p* = .0214). Compared to No + Mild NSSI, both Moderate NSSI (coeff estimate = −0.01, t(455) = −2.81, *p* = .0051) and Severe NSSI (coeff estimate = −0.01, t(455) = −2.03, *p* = .0426) groups showed different temporal cortisol trajectories (Figure 3a). Overall group differences on cortisol trajectories remained significant after including covariates (medication use, age, BDI, CTQ, and PAI-A anxiety scores) (*p* = .0079), with Severe and No + Mild NSSI groups still significantly different from each other (coeff estimate = −0.02, t(264) = −2.90, *p* = .004). Even when we excluded participants with a current or past psychiatric diagnosis and low levels of NSSI from the No + Mild NSSI group (resulting in an *N* = 20 for healthy controls), the groups showed a similar pattern (Figure S3). Linear mixed models on the observed stress and on the self-reported stress ratings did not reveal any significant temporal differences between the NSSI groups (Figures 3b and 3c).

To explore the additional variance related to past suicide attempt, we repeated the above analyses using the five NSSI/SA grouping. The results can be found in the Supplementary Materials.

### Aim 2: Examining how combinations of each of the Sustained Threat variables helps to explain the variance of NSSI severity

Multiple linear regression analyses were used to test if the multi-level Sustained Threat variables listed in Table 1 significantly predicted NSSI severity (continuous outcome: number of lifetime episodes). Stepwise model testing included all the main predictors in the most inclusive model and only the intercept in the least inclusive model. (Note: when considering all possible predictor variables, the *N* was smaller because of missing values; model selection was completed with *N* = 74). Stepwise model testing revealed that the best possible explanatory model with respect to AICs is when right and left amygdala activation, and left amygdala–mPFC RSFC are the predictors of NSSI lifetime episodes (AIC = −52.85). When a linear regression model was applied only with these three variables on the larger sample of adolescents with usable fMRI resting-state and task data (*N* = 118), the model significantly predicted NSSI lifetime episodes (adjusted R^2^ = 0.11, *F*(3,97) = 5.26, *p* = .0021). Higher NSSI lifetime total score was predicted by higher right amygdala activation (coeff estimate = 0.15, t(97) = 2.48, *p* = .0149), but lower left amygdala activation (coeff estimate = 0.16, t(97) = −2.39, *p* = .0189) and lower left amygdala–mPFC RSFC (coeff estimate = −3.14, t(97) = −2.61, *p* = .0106). When we included age, BDI, CTQ, PAI-A anxiety, and medication status as covariates, the model as a whole was still significant in predicting NSSI lifetime episodes (adjusted R^2^ = 0.29, *F*(8,56) = 4.30, *p* < .001), yet possibly due to the much smaller sample size, the effects from the brain measures were no longer significant. When we compared this brain + covariates model (AIC = 135.31) to a model including only the covariates (AIC = 130.26) we found no significant difference between them (*p* = .844).

Follow-up analysis results exploring whether the multilevel Sustained Threat variables would predict suicidal ideation (as measured by BSSI) are reported in the Supplementary Materials.

### Aim 3: Examining how the coordination between measures of Sustained Threat may vary across NSSI severity groups

**Figure 4.**
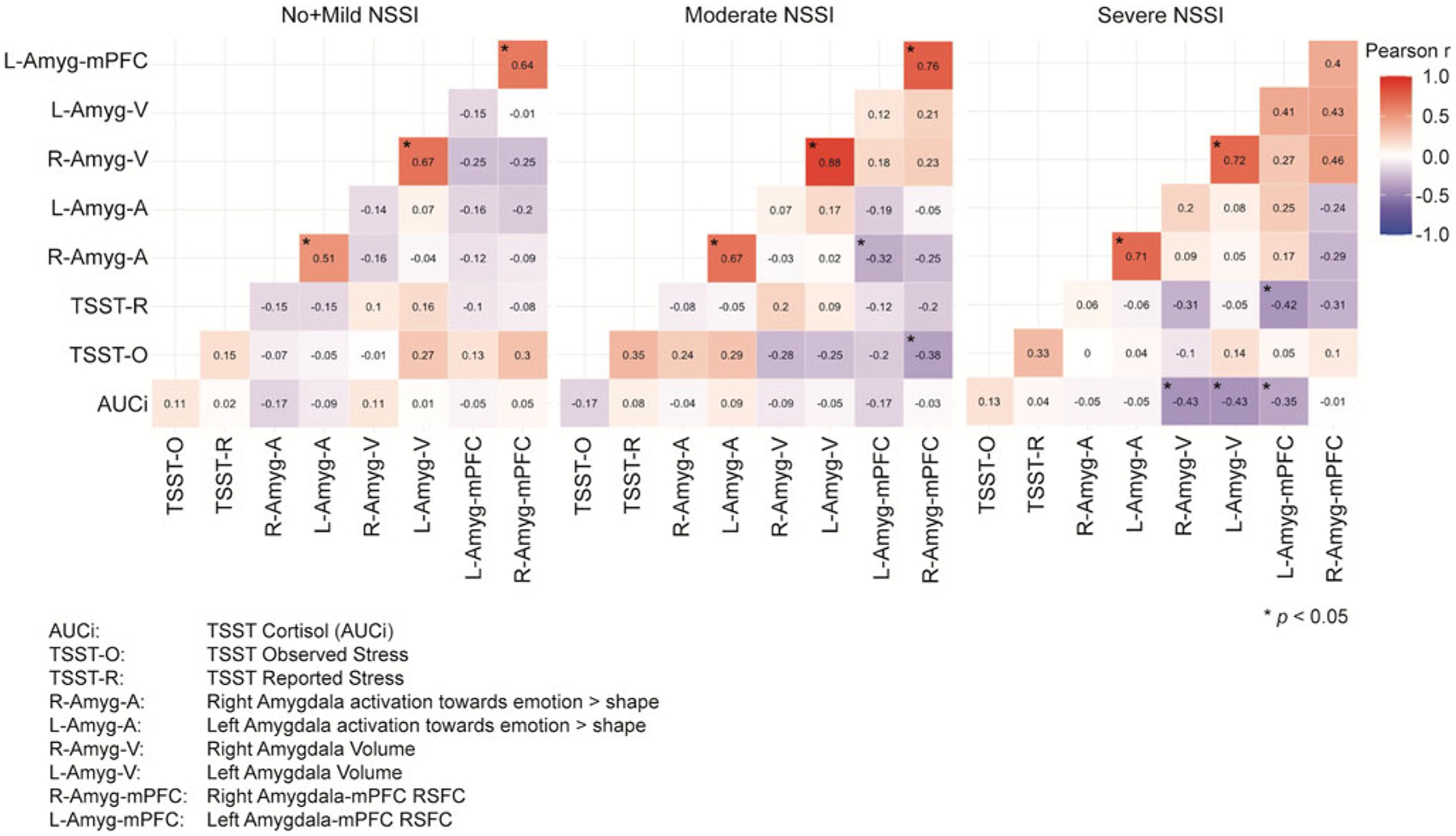
Correlation matrices of sustained threat measurements across nonsuicidal self-injury (NSSI) severity groups. Note that the significant correlations indicated with an asterisk refer only to the within group correlations, not to between group correlations.

The Jennrich test to compare the correlation matrices representing the interrelationships of our Sustained Threat variables by group revealed significant differences between No + Mild NSSI (*N* = 44) and severe NSSI (*N* = 27) groups (χ^2^ = 63.36, FDR adjusted *p* < .001), between No + Mild NSSI and moderate NSSI (*N* = 47) groups (χ^2^ = 83.90, FDR adjusted *p* < .001), and between moderate and severe NSSI groups (χ^2^ = 128.58, FDR adjusted *p* < .001) (Figure 4).

**Figure 5.**
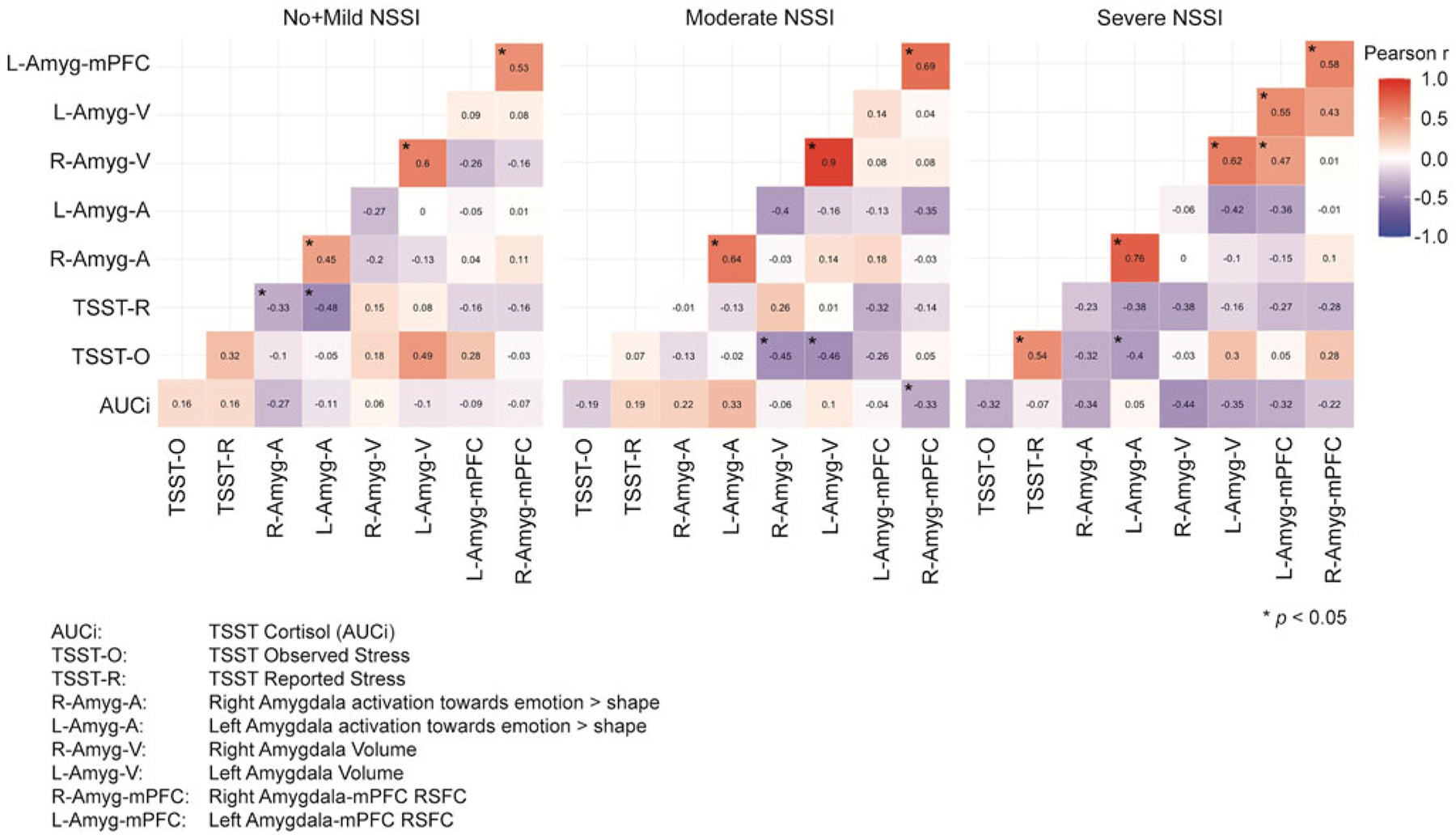
Correlation matrices of sustained threat measurements across nonsuicidal self-injury (NSSI) severity groups after adjusting the variables for the covariates. Note that the significant correlations indicated with an asterisk refer only to the within group correlations, not to between group correlations.

The patterns of correlations between the threat measures are quite different across groups, following the general pattern of stronger between-variable relationships corresponding to greater NSSI severity. To follow up on these results, we tested whether these significant differences stand when all the variables are controlled for age, BDI, CTQ, PAI-A anxiety, and medication status. Jennrich correlation matrices comparisons on these adjusted variables revealed even stronger significant differences between No + Mild NSSI (*N* = 44) and Severe NSSI (*N* = 27) (χ^2^ = 89.19, FDR adjusted *p* < .001), between No + Mild NSSI and Moderate NSSI (*N* = 47) (χ^2^ = 123.43, FDR adjusted *p* < .001), and between Moderate NSSI and Severe NSSI (χ^2^ = 314.89, FDR adjusted *p* < .001) (Figure 5).

Follow-up analyses tested how the Sustained Threat measures integrated differently across groups defined by NSSI/SA groups. They were all found to be significantly different from each other. The results and the related figures (Figures S2 & S3) can be found in the Supplementary Materials.

## Discussion

In this study we implemented an RDoC approach to examine neurobiological functioning in adolescents with NSSI. Specifically, we conducted a multilevel (brain structure and function, physiological response, behavior, self-report) assessment of the RDoC construct of Sustained Threat within the Negative Valence domain, which has been implicated in NSSI (Westlund Schreiner et al., 2015 Westlund Schreiner et al., 2017a). Key strengths of this work include the multiple levels of analysis approach, the integrative analyses, and the utilization of a transdiagnostic sample exhibiting a range of NSSI severity and other clinical symptoms.

One of the primary Sustained Threat systems that we interrogated here was the physiological stress response (Aim 1). Here we report a blunted pattern of physiological stress response (despite elevated self-reported and observed stress) in adolescents with severe NSSI, replicating and extending previous work by our group (Klimes-Dougan et al., 2019), and others (Kaess et al., 2012; Plener et al., 2017). This failure to rally self-preservatory resources could reflect allostatic changes that take place in the context of chronic stress. It may be that the physiological stress systems in adolescents with severe NSSI have undergone “wear-and-tear”, resulting in allostatic overload. Allostatic overload takes place when the body’s physiological systems are overused; accumulated stress over time results in a system imbalance, leading to hyper- or hypo-production of mediators such as cortisol, such that the system is no longer optimally adaptive or protective (McEwen, 2005; McEwen & Wingfield, 2003). This could be related to individual differences represented by adolescents in the Severe NSSI group, who perhaps may be more reactive to threat cues in their environment, leading to chronically low cortisol response, even in the face of threat (and self-reported high stress). Another potential explanation is that repeated engagement in NSSI may *itself* exacerbate the wear-and-tear on the body, accentuating the blunted response. Interestingly, in contrast to Severe NSSI’s blunted cortisol response, Moderate NSSI showed a more adaptive physiological response pattern (to a similar degree as No + Mild NSSI but about 15 minutes earlier), followed by recovery. This potentially suggests that earlier on in the course of NSSI, the HPA response shows early signs of dysregulation but at that stage is not yet showing downregulation of cortisol response. We cannot tell from this cross-sectional data whether these adolescents in the Moderate NSSI group would go on to show a blunted physiological response in the context of more persistent NSSI; only future longitudinal work can examine questions related to how HPA patterns evolve over time in the context of chronic stress, allostatic change, development and intervention.

To assess the “circuit” level of Sustained Threat, we used multimodal neuroimaging approaches to measure amygdala volume, amygdala activation in response to threat, and amygdala–mPFC connectivity at rest. When these levels of analysis were analyzed separately as (Aim 1), results showed similar amygdala volumes across groups, lower bilateral amygdala–mPFC RSFC in Severe NSSI (which was still significant on the right after correcting for covariates), and greater right amygdala activation in Moderate NSSI (which was no longer significant after correcting for covariates). These findings add to prior neuroimaging research relevant to Sustained Threat in adolescents with NSSI. With respect to amygdala RSFC, our group’s prior work (Westlund Schreiner et al., 2017b) and others (Santamarina-Perez et al., 2019) have found evidence for lower amygdala–frontal RSFC in adolescents with NSSI. Here we extend this by showing that these abnormalities are most evident in those with more severe NSSI. Given this is cross-sectional data, the current findings cannot tell us if having low amygdala–RSFC predisposes to developing severe and chronic NSSI, or if low amygdala–RSFC is an adaptive/allostatic change that occurs in the context of chronic distress and chronic NSSI. With respect to amygdala activation to threat, while our prior study did not find group differences between adolescents with NSSI and healthy controls (Westlund Schreiner et al., 2017b), two other studies reported NSSI-related enhanced amygdala activation during a negative emotion task (Plener et al., 2012) and self-administered painful stimuli (Osuch, Ford, Wrath, Bartha, & Neufeld, 2014). Discrepancies across studies may stem from variance across samples in NSSI severity: our finding that Moderate, but not Severe NSSI showed elevated amygdala activation could suggest that with time, like the cortisol responses, initially elevated amygdala responses also undergo allostatic processes and become attenuated. This speculation requires testing in a longitudinal study.

In addition to examining how each level of analysis related to NSSI severity, we also conducted multiple linear regression analyses, to combine multiple levels of analyses in predicting NSSI severity (Aim 2). This strategy may hold promise for providing a more complete and dimensionally oriented picture of how atypical patterns of Sustained Threat may be represented in adolescents with NSSI. After model selection in which all nine sustained threat variables were considered, we found that lower amygdala–mPFC RSFC, higher right and lower left amygdala activation best predicted greater lifetime NSSI episodes. This approach allowed us to see the pattern (which was not apparent with single-level analyses) that not only was *higher right* amygdala activation towards threatening stimuli relevant to NSSI severity, but *lower left* amygdala activation was related to NSSI. Although our follow-up analyses suggested that when considering relevant covariates, these specific brain measures were no longer significant in predicting NSSI severity, it is notable that the covariate analyses were limited by smaller sample sizes; we may have been underpowered to fully confirm these complex multilevel brain models when also considering multiple relevant covariates, let alone test interactions. Given the unlikelihood that a single brain, biological or behavioral measure would fully account for a complex behavior such as NSSI, capturing the intersectionality of these multifactorial mechanisms will require multimethod approaches with sufficient sample sizes for each method to confer adequate power.

Another integrative approach taken here was to examine the correspondence between the different levels of analysis of Sustained Threat, and compare correlation matrices representing the interrelationships between these nine measures across NSSI severity groups (Aim 3). Patterns of correspondence across systems may represent an efficient use of resources that are orchestrated in a coordinated way to achieve a goal (Cicchetti & Toth, 2009), particularly in the context of threat when immediate action may be needed to preserve personal safety. It is likely that unfettered coordination between systems may characterize dysfunction (Saarni, 1988). For example, in some contexts, emotional dissemblance (separating internal experience from external expression) is optimal (e.g., avoiding yelling at a boss even though one is upset with them). The results of this study revealed an intriguing pattern in which intermeasure relationships (both negative and positive correlations) were stronger in Severe NSSI than for the other two groups. This matrix approach only allows us to speculate about the nuances in the different correspondence patterns, including that adolescents in Severe NSSI (but not the other groups) show a negative association between amygdala–mPFC RSFC and cortisol responses to stress. These results build on our previous findings (Thai et al., 2020), perhaps suggesting that under risk conditions (depression and NSSI), across-level associations differ from adolescents with low levels of psychopathology. A pattern of limbic activation within the context of strong emotion has been positively associated with cortisol under both high and low risk conditions (Cunningham-Bussel et al., 2009; Klimes-Dougan et al., 2014), but this pattern was only shown for the Moderate NSSI group in this study. In addition, one of the patterns in this study linking self-reported or observed stress responses negatively with brain connectivity (across all groups) and positively with brain activation (only for the Moderate NSSI group) was novel and intriguing. Some inconsistencies with past research should also be noted. We did not replicate our past work (Klimes-Dougan et al., 2014) showing a positive link between amygdala volume and cortisol but instead found a negative link for the Severe NSSI group, although these differences could be attributed to sample differences in cortisol summary measures (AUCg vs. AUCi) and reference groups (i.e., healthy controls versus the No + Mild NSSI here included adolescents with psychopathology). Nevertheless, these results begin to paint a picture of how the multiple arms of the threat response may be converging or diverging in adolescents with varying levels of NSSI. Namely, in the context of chronic stressors, as NSSI is increasingly used as a maladaptive coping tool, allostatic changes may take place which increasingly disconnect the different arms of the threat response system, potentially serving to minimize deleterious impact of chronic biological threat activation.

While this study was geared towards understanding the neurobiology of NSSI, the dataset presents a unique opportunity to explore how Sustained Threat correlates may also be relevant to suicide in this high-risk sample. NSSI is a key risk factor for future SAs, and some of the same neurobiological systems have been implicated in both NSSI and suicide. For example, it has been proposed that vulnerability to suicide may reflect failures of the HPA system to appropriately respond during stressful conditions (Miller & Prinstein, 2019). Relatedly, cortisol reactivity to stress has been shown to predict future suicidal ideation in adolescents (Shalev et al., 2019). A recent review of the neurobiological findings associated with NSSI and suicidity reported some convergence between suicidality and NSSI results, which complicates the ability to differentiate neurobiological alterations specific to NSSI versus suicidality (Auerbach, Pagliaccio, Allison, Alqueza, & Alonso, 2021). Our results (see Supplementary Materials) are consistent with Klimes-Dougan et al. (2019) who found that those with a history of both NSSI and SA had the lowest levels of cortisol in responses to a stressor. Our exploratory multiple regression analysis on BSSI suggested a role for amygdala volume in predicting suicidal ideation which had not been found for predicting NSSI severity. Our NSSI/SA group analyses showed that history of SA was associated with some accentuated patterns as the ones observed in association with NSSI severity. For example, history of suicide attempt(s) was associated with greater degrees of self-reported stress and observed stress, and with lower amygdala–mPFC RSFC. Furthermore, the Sustained Threat matrix comparisons of the NSSI/SA groups showed highly divergent patterns. In Severe NSSI without SA, lower AUCi correlated with higher observed stress and higher amygdala activation, while in Severe NSSI with SA, higher cortisol correlated with higher observed stress and amygdala activation. In contrast, in Moderate NSSI, observed stress and AUCi were negatively correlated only for those with a history of SA. Although these analyses have limitations due to lower power with the smaller groups, and the challenge in disentangling NSSI from suicidality just based on past SA, these findings illustrate the utility of evaluating suicide risk in the context of a sample with NSSI, a population with high co-occurrence of suicidal thoughts and behaviors, to parse out the neurobiological profiles of each of these behaviors.

A major challenge that arises in the study of neural correlates of NSSI and suicidality is the fact that these problems commonly co-occur with other forms of psychopathology, most commonly internalizing problems such as depression and anxiety, and in those with a history of trauma. Some of the same neurobiological systems (including Sustained Threat) have been implicated across depression (Henje Blom et al., 2016), anxiety (Guyer, Masten, & Pine, 2013), NSSI (Westlund Schreiner et al., 2015), suicidality (O’Connor, Gartland, & O’Connor, 2020), and trauma (Heim & Nemeroff, 2001; Pine, 2003), and since these phenomena commonly co-occur, it becomes difficult (and perhaps impossible) to disentangle the effects. Our approach here was to recruit a transdiagnostic sample with a range of NSSI severity, to optimize our ability to measure relationships between dimensional measures of Sustained Threat with NSSI severity. In a departure from prior work by our lab and others (Reichl et al., 2016; Westlund Schreiner et al., 2017a, 2017b) we did not include a “healthy control” group for comparison. While there was a subgroup of the No + Mild NSSI group that had no psychiatric diagnosis and no NSSI (see Figure S3 in the Supplements), the primary approach for this study was to examine the larger No + Mild NSSI group including adolescents both with and without comorbid psychiatric disorders who had fewer than four episodes of NSSI. While this strategy to some extent mitigated the confound of NSSI being correlated with depression and anxiety, we did still have greater levels of psychopathology in the more severe NSSI groups. Thus, our secondary approach was to conduct follow-up analyses to test if the relationships between NSSI severity and Sustained Threat measures were still significant after correcting for age, depression, anxiety, medication status, and CTQ scores. Since the number of subjects with complete data on all measures was much smaller, our strategy was to report results examining NSSI severity effects both with and without covariate correction. Results which withstood correction for covariates included the lower amygdala–mPFC RSFC in severe NSSI, differences in cortisol reactivity patterns between NSSI severity groups, and the group differences on interrelationships between Sustained Threat measures.

Although our analyses examining how cortisol reactivity differs across NSSI groups did withstand correction for CTQ scores, it is still important to consider how effects may be partially explained by early adverse experiences. In this sample, the Moderate NSSI group reported greater levels of trauma exposure than the Severe and No + Mild NSSI groups. Previous studies have demonstrated blunted cortisol secretion in individuals who have experienced severe acute and chronic stress (Heim, Ehlert, & Hellhammer, 2000; Gunnar & Vazquez, 2001), including child abuse (Bunea, Szentágotai-Tătar, & Miu, 2017) and bullying by peers (Ouellet-Morin et al., 2011). However, the evidence is somewhat mixed, as both hyper- and hypocortisol reactivity have been shown in populations who have experienced trauma or maltreatment (Gunnar & Donzella, 2002; Lupien et al., 2009). Adding to this complexity, our Moderate NSSI group, who had the highest CTQ scores, did not show a blunted cortisol response. While our follow-up analyses controlled for CTQ, this retrospective measure may not always be successful in fully capturing an adolescent’s prior adverse experiences (see Baldwin, Reuben, Newbury, & Danese, 2019, for a review on discordance between prospective and retrospective measures of child adversity). In short, the impacts from previous adverse experiences may well be a driving factor in some of the findings reported here.

### Limitations

The findings reported here should be considered in the context of several study limitations. First, the multimodal approach used here is not only a strength but also a liability, in that it significantly increases the risk for missing data. To collect data on all these levels of analysis, our study protocol required three different baseline visits. Having multiple visits increases the risk for dropout, especially in families experiencing significant stress. Also, some of the participants found the measures to be time-consuming and/or emotionally taxing, and chose not to complete all of them. While the original sample size for our project is substantial, there was missingness distributed differently across the different types of data, such that analyses combining all types of data had considerably lower participant numbers. Furthermore, the COVID-19 pandemic further interrupted participant recruitment and data collection, adding to the stress of families, curtailing our in-person TSST visits, and introducing a gap between the clinical and MRI assessments.

Several issues pertaining to external validity and sampling bias should be considered with this sample. The results may not be relevant to males because the study only included adolescents who were assigned female sex at birth. Although we intended to recruit a diverse sample of adolescents who would be representative of the general population, our final sample had relatively low rates of racial and ethnic minorities, and was also above average on socioeconomic status. Furthermore, an especially at risk group for NSSI and other self-injurious thoughts and behaviors is sexual and gender minorities (Liu et al., 2019); while the online version of the KSADS (KSADS-COMP) does now collect information regarding sexual orientation and gender identity, earlier versions of this measure had an error where the data for 69 participants was unusable, and 38 of our earlier participants completed the paper version (KSADS-PL) that did not include questions about sexual orientation or gender identity preventing systematic analyses. These qualities of the representativeness of our sample further limit the external validity of our findings.

Regarding sampling bias, we focused our recruitment on parents of adolescents with NSSI; this required parents to know about their child’s NSSI prior to responding to our advertisements. This strategy under-represents adolescents who have kept their NSSI a secret from their parents. Thus, our sample is biased towards adolescents who are likely to share with their parents their NSSI, many of whom were already receiving treatment. Along these lines, although we had initially hoped to focus primarily on younger adolescents (e.g., age 12–14 years) who were in the early stages of adolescent development and NSSI engagement, the sample was somewhat biased towards older adolescents due to multiple factors including parent concerns about participant burden and the greater likelihood of having braces in the earlier teen years. Furthermore, since teens tend to hide their NSSI behavior from their parents, we are more likely to hear from parents with older teens (after NSSI has been present for some time). This also likely negatively impacted our ability to recruit adolescents into the “Mild” category. There is likely to be inherent bias in the self-reports of past NSSI episodes, where some adolescents under-report and others over-report. While our analyses attempted to address co-occurring symptomatology such as depression and anxiety, and other likely contributory factors such as medication status and past trauma, we focused on factors most likely to be directly relevant to Sustained Threat, and did not consider all forms of psychopathology (e.g., impulsivity that may also be highly relevant to engaging in self-harm (Beauchaine, Hinshaw, & Bridge, 2019).

Finally, a key study limitation is the cross-sectional nature of the data analyzed here. The findings raise several questions that we cannot answer here: when did these aberrant patterns of Sustained Threat emerge? Did they result from early adverse experiences? Are these biological traits that are present early in development which predispose a young person both to developing NSSI and suicide risk? Do they emerge after the onset of NSSI, and set the stage for a future suicide attempt? Do these patterns reflect an allostatic response to chronic stress, as an adaptive biological strategy of ignoring/dampening toxic responses to persistent threat signals? While we found some intriguing patterns with respect to how Sustained Threat measures may become dis-connected over time in the context of chronic stress and ongoing NSSI engagement, this interpretation remains as a hypothesis until it can be confirmed with data showing within-person change over time. This longitudinal research is currently underway with the aim of understanding how the multiple layers of the Sustained Threat system evolve alongside clinical trajectories over the course of adolescents in youth with NSSI.

## Conclusion

NSSI is a disordered behavior that is increasingly common in adolescents and represents a critical risk factor for future suicide attempts. A multilevel analysis of the threat system found that physiological blunting of the cortisol stress response was exhibited in those with severe NSSI, and that characterization of threat circuitry is most informative when multiple neuroimaging modalities are used. Integrative analyses suggest that much can be learned from examination of how the different levels of analysis of the threat system relate to each other, with patterns of interrelationships of these measures changing significantly with respect to NSSI severity. Overall, the findings underscore the complexity of neurobiological abnormalities in NSSI, and the importance of examining the orchestration between systems to better understand these neurobiological mechanisms during development. Continued efforts to more fully explore these preliminary findings while accounting for co-occurring problems (e.g., suicide risk, depression, anxiety) and processes (e.g., trauma) is warranted. Presently, there is a substantial dearth of treatment options that effectively treat NSSI and suicidal thoughts and behavior. The complexity of our results may underscore the potential need for multiple, different treatment strategies that can target these neurobiological mechanisms. Future work incorporating longitudinal designs (both naturalistic developmental and interventional studies) will enhance our understanding of mechanisms of change, thereby informing the development of individualized treatment.

## Supplementary Material

2

## Figures and Tables

**Table 1.** Multilevel sustained threat variables assessed in the current analysis

Brain	
*Structure*	Bilateral amygdala volume
*Function*	Bilateral amygdala activation during emotion matching task (towards emotion > shape condition)
*Connectivity*	Bilateral amygdala-mPFC resting state functional connectivity
**Physiology**	Salivary cortisol (TSST; cortisol collected at 5 different time points)
	Cortisol area under the curve with respect to increase (TSST; AUCi)
**Behavior**	Observed stress (TSST)
**Self-report**	Reported stress (TSST)

mPFC = medial prefrontal cortex

TSST = Trier Social Stress Test

AUCi = area under curve with respect to increase

**Table 2.** Demographic and clinical characteristics of Brain Imaging Development of Girls’ Emotion and Self (BRIDGES) participants grouped by lifetime nonsuicidal self-injury (NSSI) severity. For categorical variables, data are reported with sample size (*N*), and percentage in parentheses while for continuous variables data are reported as mean, and standard deviations in parentheses

	No/Mild NSSI (*N* = 53)	Moderate NSSI (*N* = 53)	Severe NSSI (*N* = 36)	Overall (*N* = 142)
*Demographic*				
Age	14.21 (1.28)	14.53 (1.35)	14.83 (0.88)	14.49 (1.24)
Race, *N* (%)				
White	42 (79.25%)	37 (69.81%)	33 (91.67%)	112 (78.87%)
More than one race	6 (11.32%)	10 (18.87%)	2 (5.56%)	18 (12.68%)
Asian	2 (3.77%)	1 (1.89%)	1 (2.78%)	4 (2.82%)
Black/African American	2 (3.77%)	1 (1.89%)	0 (0.00%)	3 (2.11%)
Other race	1 (1.89%)	2 (3.77%)	0 (0.00%)	3 (2.11%)
American Indian/Alaska Native	0 (0.00%)	2 (3.77%)	0 (0.00%)	2 (1.41%)
Hispanic/Latino, *N* (%)	6 (11.32%)	8 (15.09%)	1 (2.78%)	15 (10.56%)
Gross Income per year, *(N = 136)*				
$0,000–$24,000	2 (3.77%)	3 (6.00%)	4 (12.12%)	9 (6.62%)
$25,000–$39,999	7 (13.21%)	8 (16.00%)	0 (0.00%)	15 (11.03%)
$40,000–$59,999	2 (3.77%)	6 (12.00%)	3 (9.09%)	11 (8.09%)
$60,000–$89,999	6 (11.32%)	8 (16.00%)	8 (24.24%)	22 (16.18%)
$90,000–$179,999	26 (49.06%)	16 (32.00%)	8 (24.24%)	50 (36.76%)
Over $180,000	10 (18.87%)	9 (18.00%)	10 (30.30%)	29 (21.32%)
*Clinical*				
Lifetime total NSSI episodes *(N = 136)*	6.83 (46.66)	16.35 (17.62)	170.11 (238.37)	52.21 (141.87[BKD2])
Taking psychiatric medications *(N = 138)*	11 (20.75%)	34 (68.00%)	27 (77.14%)	72 (52.17%)
BDI-II *(N = 133)*	6.84 (8.43)	22.04 (13.62)	22.94 (11.82)	16.44 (13.63)
BSSI *(N = 137)*	1.08 (3.58)	8.42 (9.18)	9.37 (7.74)	5.87 (8.03)
CTQ total *(N = 127)*	32.14 (12.95)	43.62 (12.26)	38.74 (11.12)	37.69 (13.17)
PAI-A anxiety *(N = 102)*	49.61 (10.74)	63.26 (10.91)	69.78 (12.68)	59.25 (13.90)
Taking psychiatric medications *(N = 138)*	11 (20.75%)	34 (68.00%)	27 (77.14%)	72 (52.17%)
Lifetime suicide attempt, *(N = 139)*	3 (5.66%)	20 (40.00%)	21 (58.33%)	44 (31.65%)

NSSI = nonsuicidal self-injury

BSSI = Beck Scale for Suicidal Ideation

BDI-II = Beck Depression Inventory II

CTQ = Child Trauma Questionnaire

PAI-A = Personality Assessment Inventory Adolescent

**Table 3.** The correlations between all of the variables used in the current study

Measurements	TSST cortisol (AUCi)	TSST observed	TSST reported	Right amygdala Activation	Left amygdala Activation	Right amygdala Volume	Left amygdala Volume	Left amygdala-mPFC RSFC	Right amygdala-mPFC RSFC	BDI-II	CTQ	Age	Medication	PAI-A anxiety	BSSI	NSSI
TSST Cortisol (AUCi)	1															
TSST Observed	−0.01	1														
TSST Reported	0.18	0.29	1													
Right Amygdala Activation	−0.15	0.01	−0.19	1												
Left Amygdala Activation	0.02	−0.05	−0.32*	0.4*	1											
Right Amygdala Volume	−0.03	−0.07	0.09	−0.12	−0.26	1										
Left Amygdala Volume	0.03	0	−0.04	−0.04	−0.03	0.82****	1									
Left Amygdala-mPFC RSFC	−0.16	−0.15	−0.15	0.02	0	0.05	0.12	1								
Right Amygdala-mPFC RSFC	−0.08	−0.07	−0.24	0.03	0.02	0.1	0.22	0.65****	1							
BDI-II	0.07	0.31	0.21	0.26	−0.11	−0.12	−0.21**	−0.23**	−0.29***	1						
CTQ	0.06	0.18	0.12	−0.03	−0.07	−0.22	−0.17*	−0.35***	−0.46****	0.4**	1					
Age	0.11	−0.02	0.05	0.14	−0.14	0.06	−0.14	−0	−0.15	0.3	−0	1				
Medication	−0.02	0.22	−0.02	0.21	−0.12	−0.08	−0.12	−0.23**	−0.25**	0.53****	0.34**	0.09	1			
PAI-A anxiety	−0.03	0.2	0.15	0.12	0.07	−0.06	−0.08*	−0.18*	−0.18*	0.61***	0.2	0.29	0.42**	1		
BSSI	0.12	0.12	0.17	0.11	0	−0.16	−0.11*	−0.27**	−0.32***	0.51***	0.35**	0.07	0.25*	0.24*	1	
NSSI	−0.03	0.14	0.03	0.18	−0.06	0.14	0.03	−0.18*	−0.12*	0.5**	0.1	0.29	0.37**	0.34*	0.48**	1

**** *p* < .0001

*** *p* < .001

** *p* < .01

* *p* < .05

NSSI = nonsuicidal self-injury (lifetime total episodes)

BSSI = Beck Scale for Suicidal Ideation

BDI-II = Beck Depression Inventory II

CTQ = Child Trauma Questionnaire

PAI-A = Personality Assessment Inventory Adolescent

TSST = Trier Social Stress Test

AUCi = area under curve with respect to increase

Amygdala–mPFC RSFC = Amygdala–middle Prefrontal Cortex Resting State Functional Connectivity

**Table 4.** One-way analysis of variance (ANOVA) results for the nonsuicidal self-injury (NSSI) severity group differences on each variable included in this study

Measure	Main group difference *F* and *p* values	No + Mild NSSI	Moderate NSSI Coefficient estimates, *t* and *p* values	Severe NSSI Coefficient estimates, *t* and *p* values	After controlling for age, BDI, CTQ, PAI-A anxiety, and medication status
***Sustained threat variables***					
**Cortisol (AUCi)**	*F*(2,109) = 2.57 *p* = .0814	Reference	*coeff* = −*2.77* *t(109) = −1.82* *p = .0719*	**coeff** = −**3.36** **t(109)** = −**2.02** ***p* = .0463**	*F*(7,58) = 0.67 *p* = .6952
**Reported stress**	***F*(2,113) = 3.39** ***p* = .0373**	Reference	**coeff = 0.47** **t(113) = 2.07** ***p* = .0403**	**coeff = 0.58** **t(113) = 2.31** ***p* = .0229**	*F*(7,59) = 1.10 *p* = .3762
**Observed stress**	*F*(2,114) = 2.2 *p* = .1155	Reference	coeff = 0.25 t(114) = 1.33 *p* = .188	**coeff = 0.43** **t(114) = 2.05** ***p* = .043**	F(7,60) = 1.10 *p* = .3792
**Right amygdala volume**	*F*(2,130) = 0.79 *p* = .4567	Reference	coeff = −38.11 t(130) = −0.90 *p* = .370	coeff = −57.14 t(130) = −1.19 *p* = .238	*F*(7,76) = 0.81 *p* = .5818
**Left amygdala volume**	*F*(2,130) = 0.24 *p* = .7871	Reference	coeff = −20.42 t(130) = −0.58 *p* = .563	coeff = −23.86 t(130) = −0.60 *p* = .552	*F*(7,76) = 0.54 *p* = .8018
**Right amygdala task activation**	*F(2,114) = 2.86* *p = .0616*	Reference	**coeff = 0.79** **t(114) = 2.28** ***p* = .0247**	coeff = 0.67 t(114) = 1.66 *p* = .0996	*F*(7,65) = 0.89 *p* = .5202
**Left amygdala task activation**	*F*(2,114) = 0.45 *p* = .6395	Reference	coeff = 0.22 t(114) = 0.70 *p* = .484	coeff = −0.09 t(114) = −0.26 *p* = .793	*F*(7,65) = 1.11 *p* = .3659
**Right amygdala-mPFC RSFC**	*F*(2,115) = 2.53 *p* = .0841	Reference	coeff = −0.01 t(115) = −1.06 *p* = .2911	**coeff** = −**0.03** **t(115)** = −**2.25** ***p* = .0264**	***F*(7,65) = 2.99** ***p* = .0087**
**Left amygdala-mPFC RSFC**	*F(2,115) = 2.91* *p = .0586*	Reference	coeff = −0.02 t(115) = −1.69 *p* = .0931	**coeff** = −**0.03** **t(115)** = −**2.29** ***p* = .0238**	*F*(7,65) = 1.19 *p* = .3219
***Clinical and demographic variables***					
**BDI**	***F*(2,130) = 29.42** ***p* < .001**	Reference	**coeff = 15.20** **t(130) = 6.63** ***p* < .001**	**coeff = 16.10** **t(130) = 6.38** ***p* < .001**	N/A
**CTQ**	***F*(2,124) = 10.42** ***p* < .001**	Reference	**coeff = 11.47** **t(124) = 4.53** ***p* < .001**	**coeff = 6.60** **t(124) = 2.39** ***p* = .0183**	N/A
**PAI-A anxiety**	***F*(2,99) = 27.5** ***p* < .001**	Reference	**coeff = 13.65** **t(99) = 4.98** ***p* < .001**	**coeff = 20.17** **t(99) = 6.88** ***p* < .001**	N/A
**Age**	*F(2,139) = 2.87* *p = .0600*	Reference	coeff = 0.32 t(161) = 1.35 *p* = .1782	**coeff = 0.63** **t(161) = 2.38** ***p* = .0189**	N/A
**Income**	*F*(2,134) = 0.82 *p* = .4417	Reference	coeff = −0.38 t(134) = −1.15 *p* = .251	coeff = 0.02 t(134) = 0.04 *p* = .966	N/A

Significant results are embolden

Trend-level significant results are italicized

NSSI = Nonsuicidal Self Injury

SA = Suicide Attempt

BDI = Beck Depression Inventory II

CTQ = Child Trauma Questionnaire

PAI-A = Personality Assessment Inventory Adolescent

AUCi = area under curve with respect to increase
