# Supplementary material for: Multimodal assessment of sustained threat in adolescents with nonsuicidal self-injury
Source: Dev Psychopathol. Author manuscript; Available in PMC 2022 Nov 20. (PMC8898324; doi:10.1017/S0954579421000754)
Supplement: 2 [file NIHMS1714053-supplement-2.docx]

**SUPPLEMENTARY MATERIALS**

Neuroimaging Preprocessing Steps

HCP pipeline includes three structural pipelines and four functional pipelines. Structural pipelines include: *PreFreeSurfer* (undistorted structural volume spaces are produced, T1 and T2 weighted images are aligned, a bias field correction and a gradient distortion correction to correct for gradient inhomogeneities within anatomical scans are applied, and each participant’s native structural volume space is registered to MNI space), *FreeSurfer* (the volume is segmented into predefined structures, including the subcortical parcels, white and pial cortical surfaces are reconstructed, and a folding-based surface registration to surface atlas is performed), and *PostFreeSurfer* (volumetric and surface based images are produced, surface registration is applied, registered surfaces are downsampled, the final brain mask and myelin maps are created). Functional data preprocessing pipelines include: *(1) fMRIVolume.* Gradient distortions within EPI images are corrected, spatial distortions are removed, volumes are realigned to compensate for subject motion, the fMRI data is registered to the structural data, the bias field is reduced, the 4D image is normalized to a global mean, and the data is masked with the final brain mask). (2) *fMRISurface.* Timeseries are brought from the volume into CIFTI space, the data are smoothed with surface-smoothing of 2mm FWHM to regularize the mapping process). (3) *ICAFIX*. Time series are linearly detrended with a high band pass filter cutoff of 2000s and a slow roll off, time series are concatenated across scans but within run and sent through independent component analysis (ICA). The FSL tool “FIX” uses the spatial independent components to classify components as “signal” or “noise”. The resulting components are then cleaned in two separate steps. First, the components are cleaned in a “non-aggressive” manner by regressing both the good and bad component time series into the data, and then the resulting bad spatial maps are multiplied by the associated time series and subtracted from the original dataset. Second, 24 head motion confounds were calculated and used as regressors to clean up all components. Third, 24 confound time series derived from the motion estimation (the 6 rigid-body parameter time series, their backwards-looking temporal derivatives, plus all 12 resulting regressors squared) are used [(Satterthwaite et al., 2013)](https://paperpile.com/c/lGYeHg/W0mDL). The motion parameters are then regressed out of the data aggressively, as they are not expected to contain variance of interest [(Griffanti et al., 2014; Salimi-Khorshidi et al., 2014; Smith et al., 2013)](https://paperpile.com/c/lGYeHg/SrP7p+UVRAI+pnFUi). *(4)* *MSMAll*. Participant data is applied for functional alignment to a group template that is based on myelin maps, resting state fMRI network and visuotopic maps [(Robinson et al., 2014)](https://paperpile.com/c/lGYeHg/aFBzZ).

Once *ICAFIX* and *MSMAll* were complete, the concatenated time series were dissociated into their respective scans and used for subsequent analyses. Following *ICAFIX* denoising and *MSMAll* alignment, CIFTI space fMRI data (both resting-state and task) was parcellated into non-overlapping ROIs using a combination of the Glasser [(Glasser et al., 2016)](https://paperpile.com/c/lGYeHg/FKwrT) and Harvard-Oxford [(Makris et al., 2006)](https://paperpile.com/c/lGYeHg/xLgLR) atlases for the cortical and subcortical ROIs, respectively. Once parcellated, the CIFTI space data was converted to matrix form.

Follow-up analyses on NSSI groups varying by past suicide attempt

*Preliminary ANOVAs on psychological and demographic measurements.* The severe group with suicide attempt history showed the highest BDI compared to all other groups: Severe NSSI with SA > Moderate NSSI with SA > Severe NSSI without SA > Moderate NSSI without SA > No NSSI without SA. These five groups also differed significantly with respect to their CTQ scores, moderate NSSI group having the highest scores: Moderate NSSI with SA > Moderate NSSI without SA > Severe NSSI with SA > Severe NSSI without SA > No NSSI without SA. Severe NSSI group with SA history demonstrated the highest PAI-A anxiety scores compared to all other groups: severe NSSI with SA > severe NSSI without SA > moderate NSSI without SA> moderate NSSI with SA > No NSSI without SA. See statistical results in Table S2.

*One-way ANOVAs on Sustained Threat variables.* Another set of ANOVAs were conducted on each of the Sustained Threat variables separately to test differences between the 5 NSSI/SA groups. Severe NSSI with SA showed higher observed stress compared to the other groups, whereas Moderate NSSI with SA showed a trend-level difference compared to all other groups. After controlling for age, CTQ, BDI, PAI-A-anxiety, and medication status, the model lost its significance, but pairwise comparisons showed that observed stress was still significantly higher for the Severe NSSI with SA group compared to all other groups. Although the group difference was not significant for TSST reported stress, pairwise comparisons demonstrated that both Severe with SA and Moderate with SA reported higher stress compared to the other groups. However, after controlling for the aforementioned covariates this finding did not hold. No significant or trend-level NSSI/SA group differences were found for AUCi and amygdala volume before or after including the covariates. Although there were no significant main group differences for amygdala task activations, the moderate NSSI with SA group showed a trend-level significantly higher right amygdala task activation, although this result lost significance after controlling for the covariates. Another trend-level group difference was observed for right amygdala-mPFC RSFC with a significant pairwise analysis result for the Severe NSSI with SA group who showed the lowest connectivity.

After the adjustments for covariates, this model became significant for the right amygdala-mPFC RSFC. As for the left amygdala-mPFC RSFC, although the main group difference was not significant, we observed that Severe and Moderate NSSI groups with suicide history showed significantly lower left amygdala-mPFC RSFC compared to the other groups; however, these results did not hold after controlling for the covariates.

*TSST measurements over time.* Linear mixed models on the cortisol levels over five points of time did not reveal a significant group difference, when we simultaneously tested the group and group by time interactions (*p* = 0.1862), although there was a trend toward difference between No+Mild NSSI without SA and Moderate NSSI without SA (coeff estimate = 0.0096, t(378) = 1.84, *p* = 0.066) (with the latter group showing an earlier peak). When we controlled for age, BDI, CTQ, PAI-A anxiety, and medication status, the difference became significant at *p* = 0.0262. However, pairwise comparisons revealed no significant difference in temporal cortisol trajectory between NSSI/SA groups. Linear mixed effects models on the observed and self-reported stress ratings did not reveal any significant differences between the NSSI/SA groups.

*Multiple regression models on BSSI.* Stepwise model testing revealed that the best possible explanatory model with respect to AIC is when right amygdala volume, stress self-report, and AUCi are the predictors of BSSI (N = 84, AIC = 355.99). When a linear regression model was applied only with these three variables on the larger sample (N = 118), the model as a whole was found to predicted suicide ideation significantly (adjusted R^2^ =0.07, *F*(3,82)=2.99, *p* = 0.0359), yet only lower right amygdala volume was significantly predicting higher suicide ideation (coeff estimate = -.01, t(82) = -2.01, *p* = 0.0474). When we added age, BDI, CTQ, PAI-A anxiety, and medication status as covariates, the model was still significant (adjusted R^2^ =0.18, *F*(8,43)=2.44, *p* = 0.0285), yet the right amygdala volume effect was no longer significant. When we compared this model (AIC = 349.23) to a model including only the covariates (AIC = 343.72) we found no significant difference between them (*p* = 0.9376).

*Correlations between measures of Sustained Threat.* Results indicated that No+Mild NSSI without SA differed from Moderate NSSI without SA (𝛘^2^ = 102.09, FDR adjusted *p* < .001) and Severe NSSI without SA matrices (𝛘^2^ = 89.37, FDR adjusted *p* < .001). Across both Severe and Moderate NSSI, the with SA versus without SA groups differed significantly (severe: 𝛘^2^ = 136.08, FDR adjusted *p* < .001; moderate: 𝛘^2^ = 125.07, FDR adjusted *p* < .001). Furthermore, across both with SA and without SA groups, Severe and Moderate NSSI matrices significantly differed from each other (𝛘^2^ = 94.56, FDR adjusted *p* < .001) (Figure S1).

FIGURE S1 HERE

When we controlled for covariates, matrix differences remained significant: No+Mild NSSI without SA differed from Moderate NSSI without SA (𝛘^2^ = 141.62, FDR adjusted *p* < .001) and Severe NSSI without SA matrices (𝛘^2^ = 87.38, FDR adjusted *p* < .001). Within Severe NSSI group, with SA versus without SA groups differed completely ( 𝛘^2^ = 4505.34, FDR adjusted *p* = 0). Moderate NSSI with SA versus without SA groups also differed significantly (moderate: 𝛘^2^ = 132.25, FDR adjusted *p* < .001). Furthermore, across both with SA and without SA groups, Severe and Moderate NSSI matrices significantly differed from each other (𝛘^2^ = 1236.23, FDR adjusted *p* < .001) (Figure S2).

FIGURE S2 HERE

Temporal trajectories of TSST measures with a Nonpsychiatric control group

Although our main purpose in this study was to utilize a more inclusive “No+Mild NSSI” participant pool (composed of adolescents both with and without comorbid psychiatric disorders), we acknowledge that approaches comparing adolescents with NSSI to a group of Healthy Controls (no psychopathology) has been more common in the literature. Therefore, we conducted a follow-up analysis to examine temporal trajectories of stress responses utilizing a “nonpsychiatric No NSSI” group in which we excluded participants with any current or past psychiatric diagnosis and any NSSI from our No+Mild NSSI Group (resulting with N =20, who had TSST cortisol data available). We observed that this group’s pattern of temporal trajectories is very similar to that of the larger No+Mild NSSI Group, and the statistical results comparing groups were also similar.

FIGURE S3 HERE
